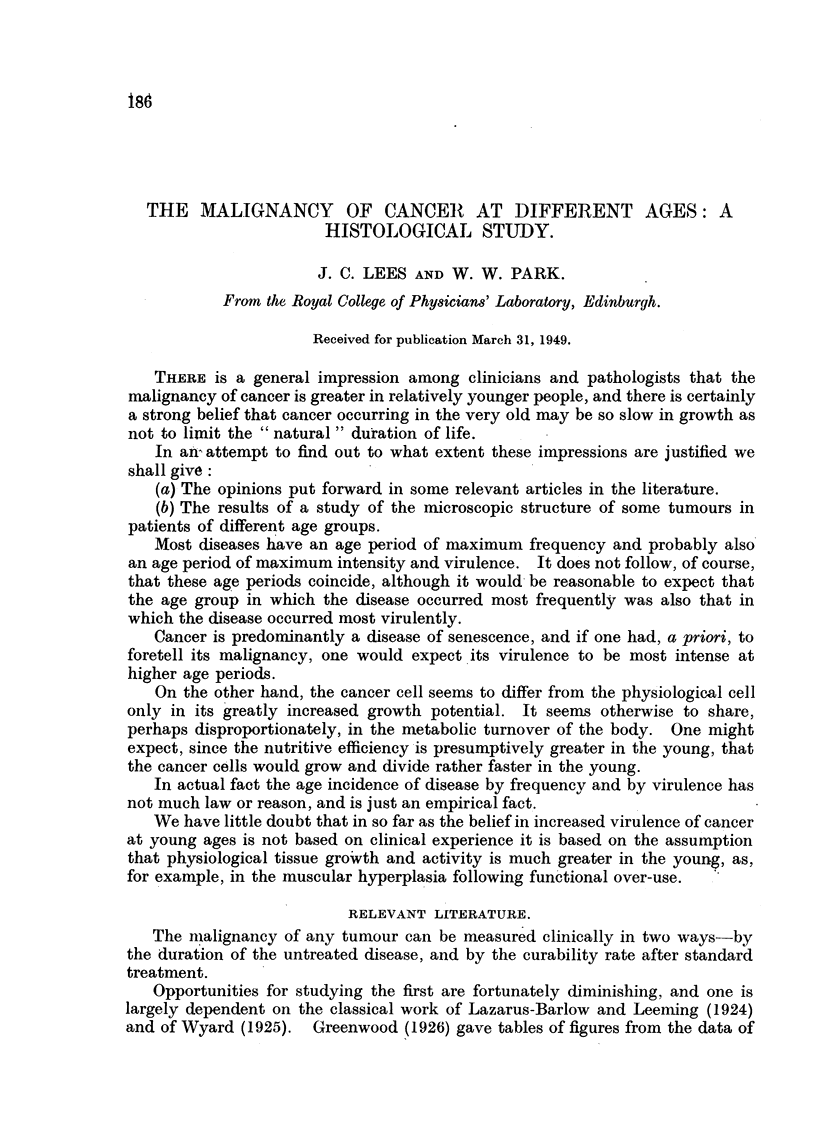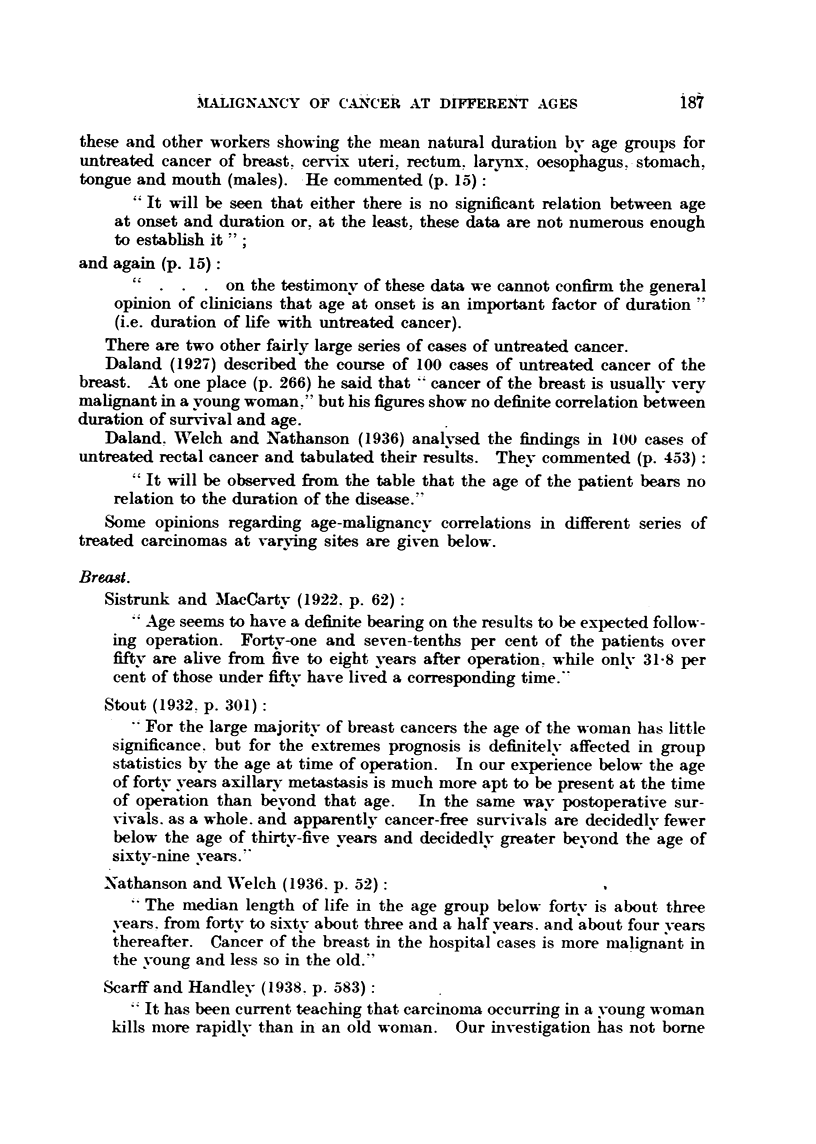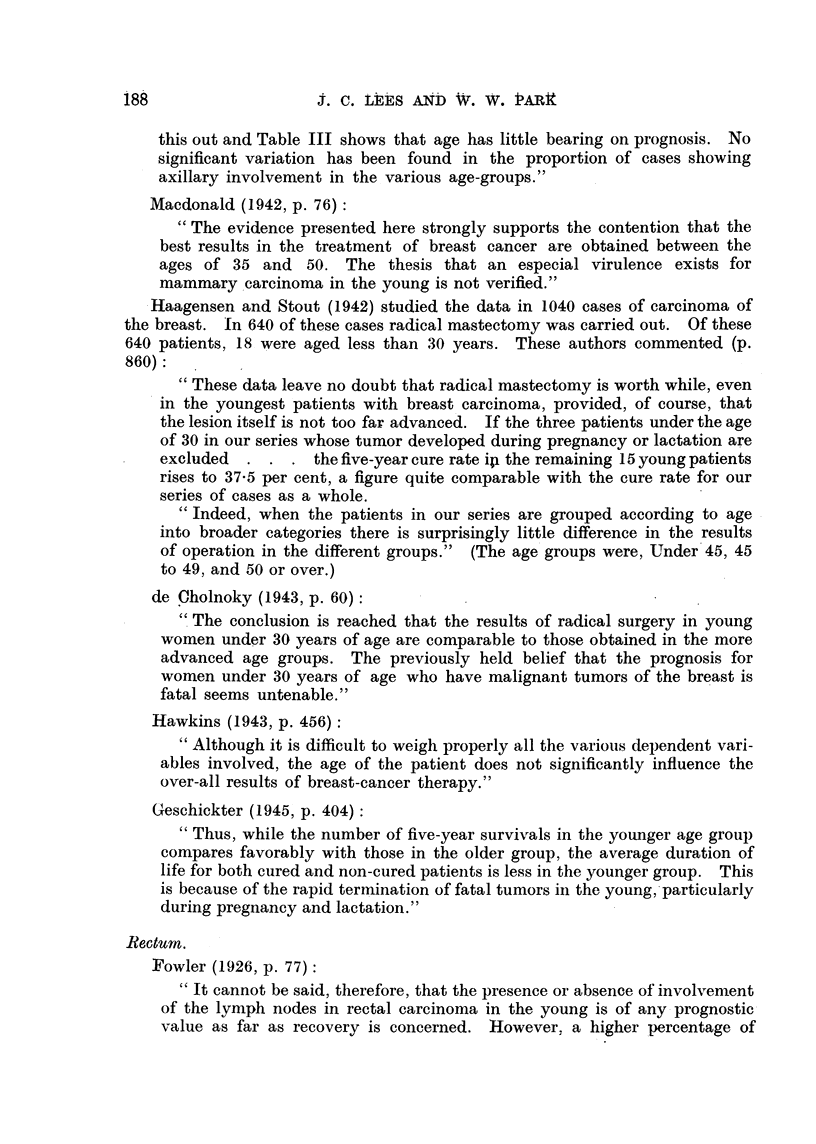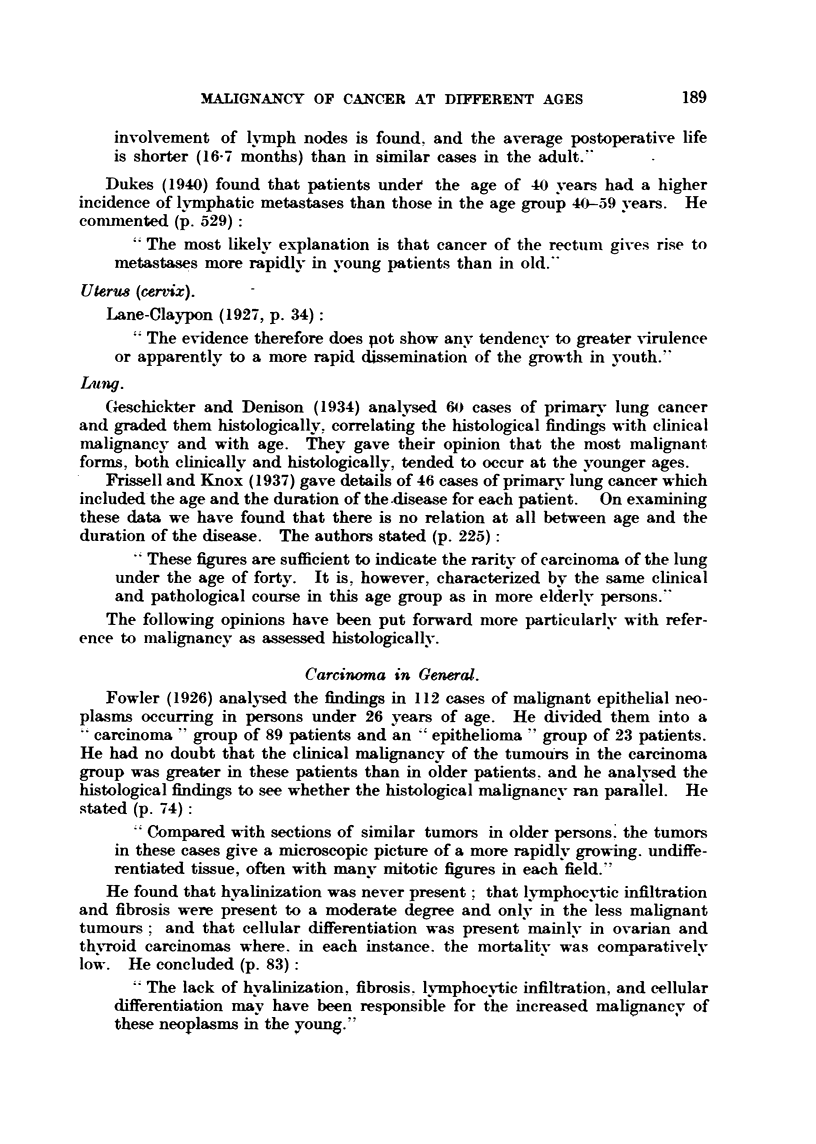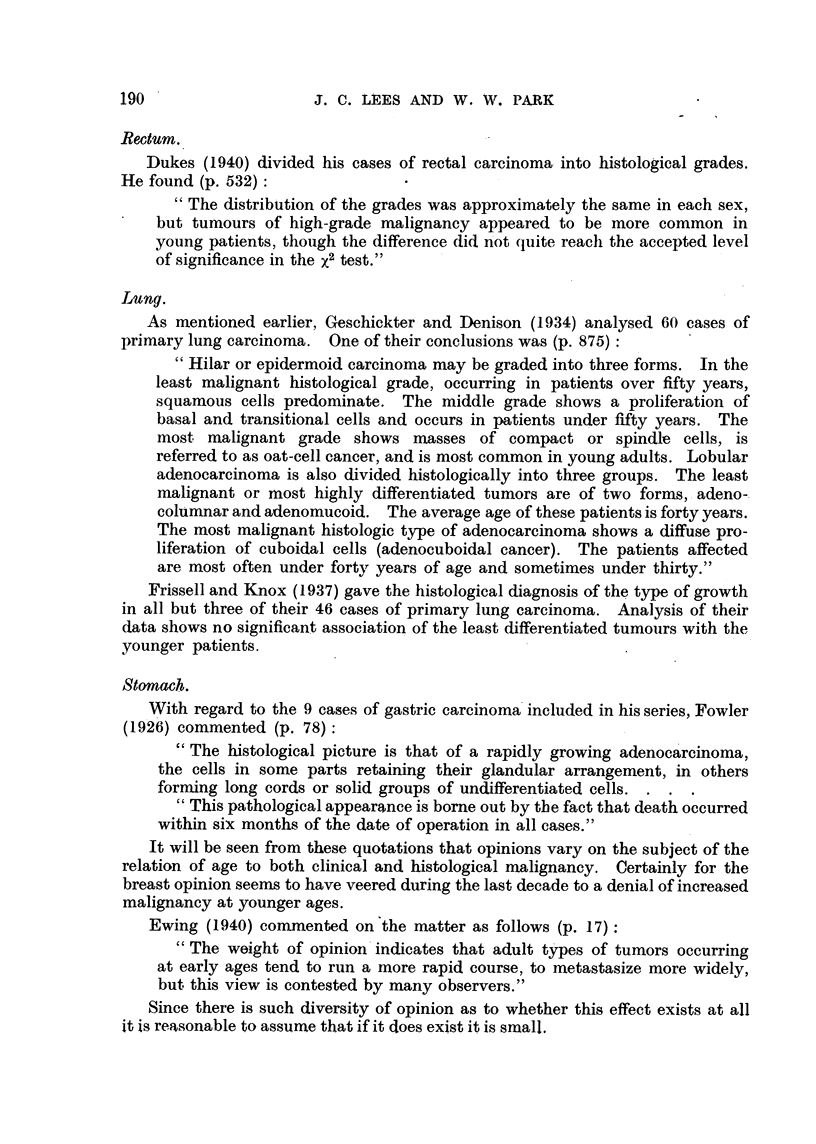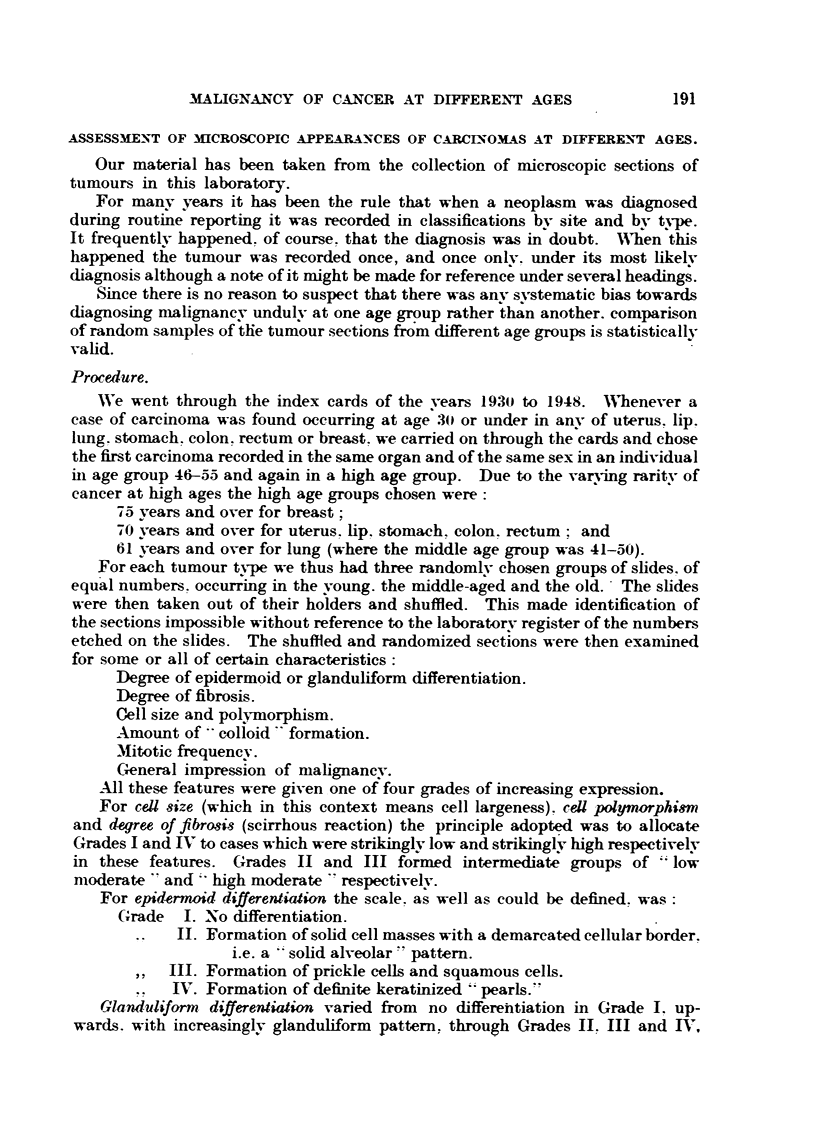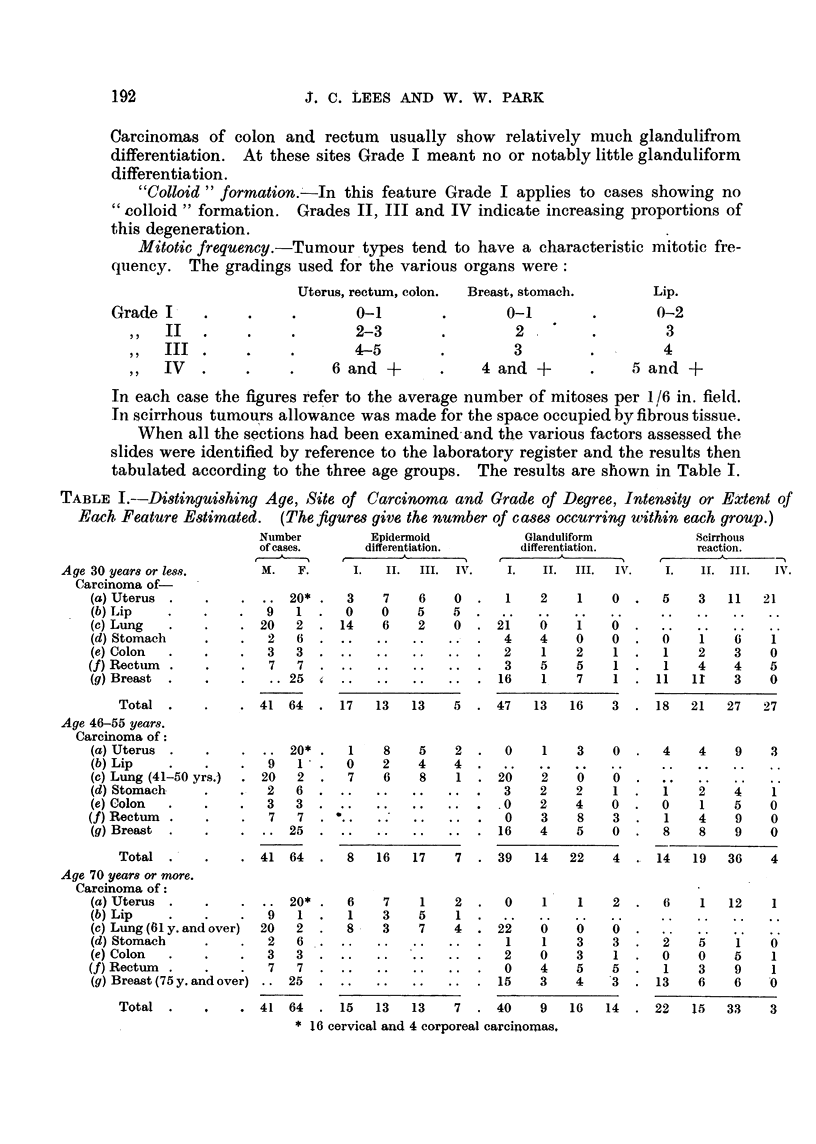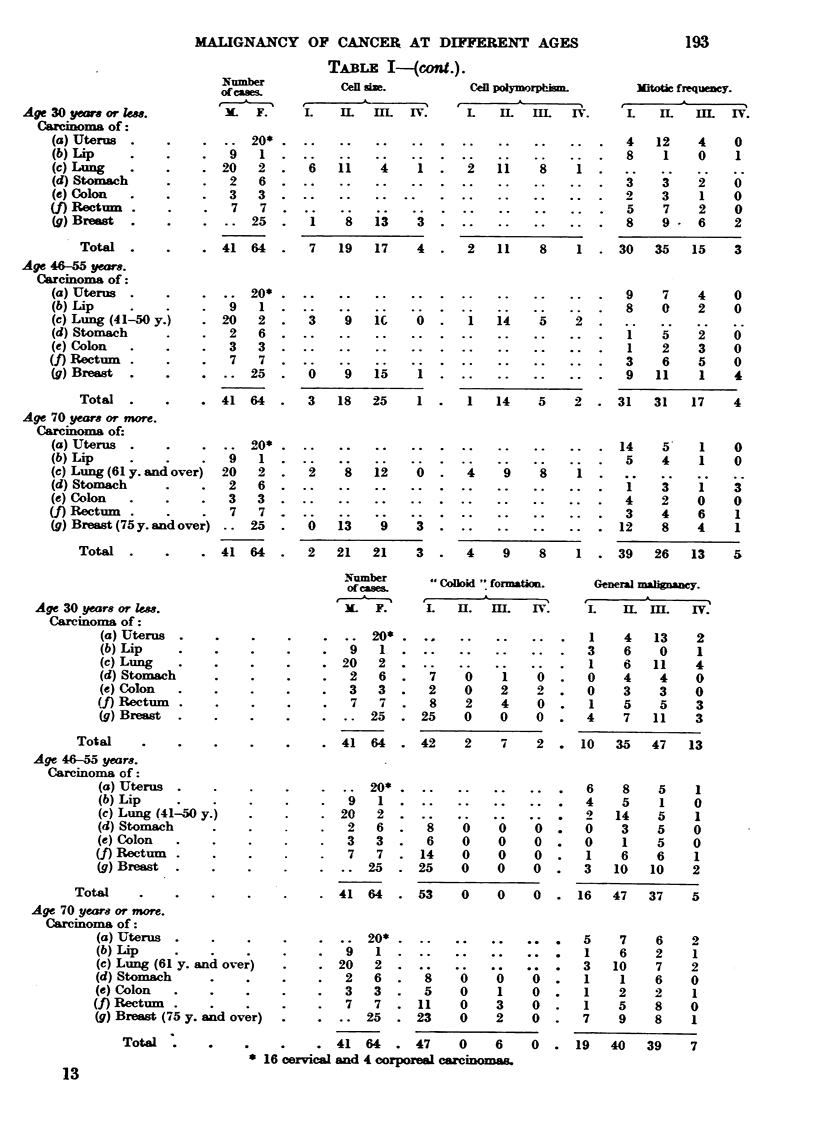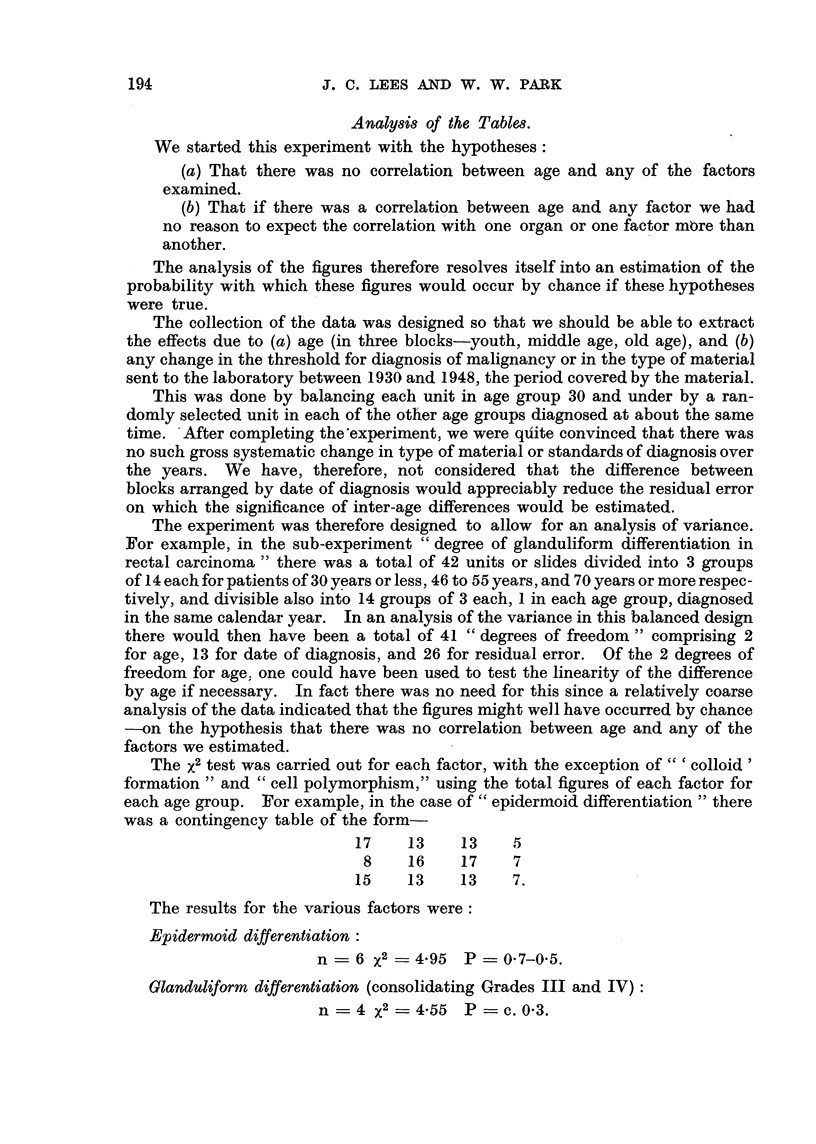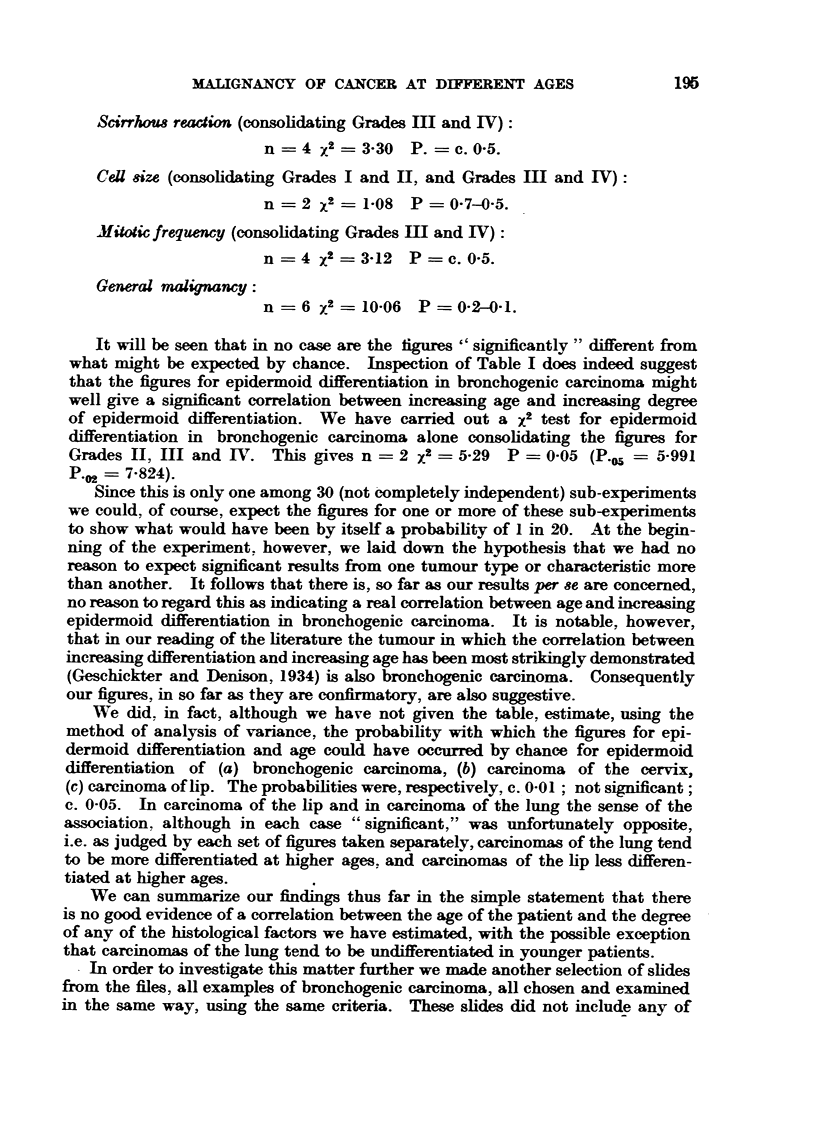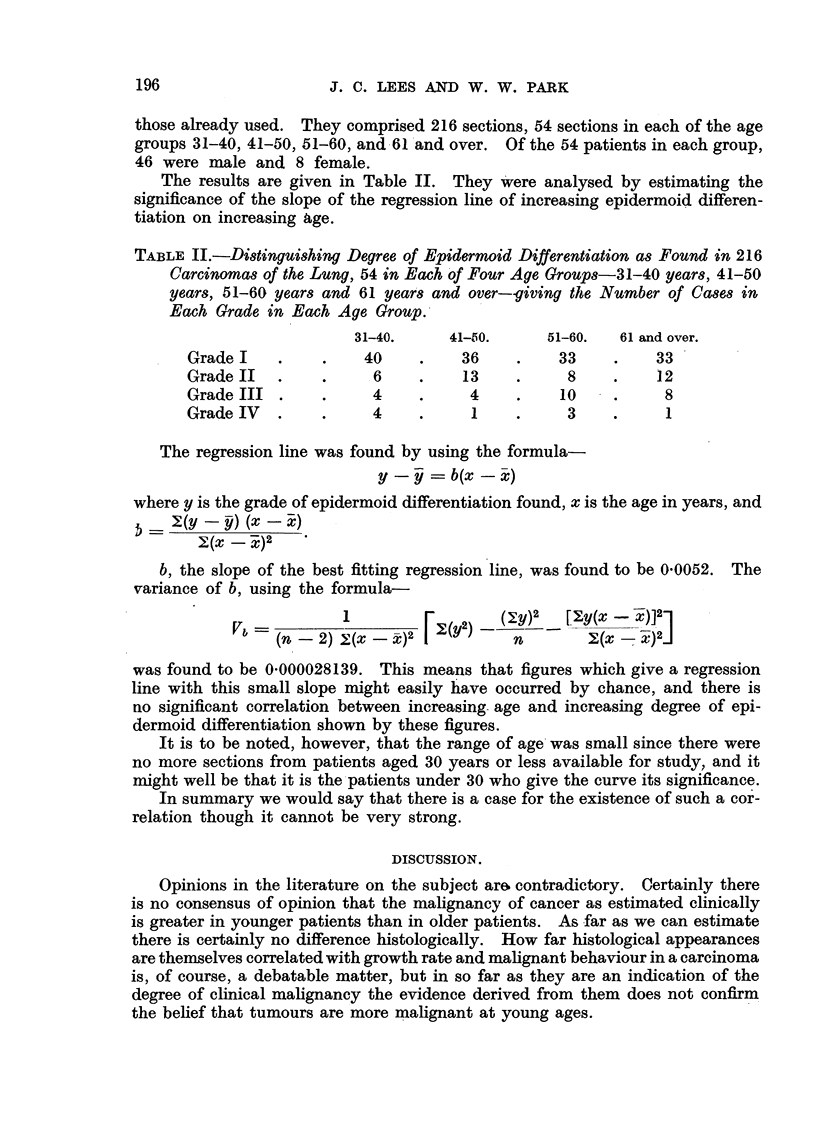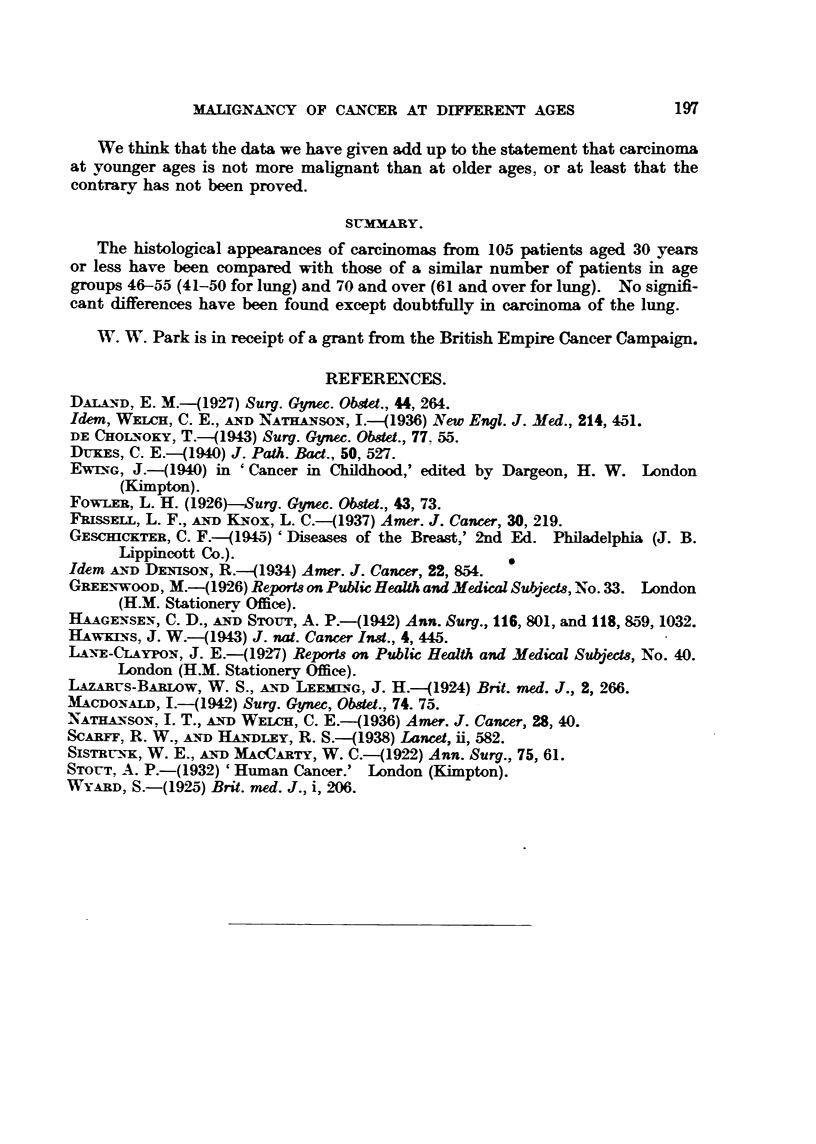# The Malignancy of Cancer at Different Ages: A Histological Study

**DOI:** 10.1038/bjc.1949.20

**Published:** 1949-06

**Authors:** J. C. Lees, W. W. Park


### THE MALIGNANCY OF CANCER AT DIFFERENT AGES: A HISTOLOGICAL STUDY.

J. C. LEES AND W. W. PARK.

*From the Royal College of Physicians' Laboratory, Edinburgh.*

Received for publication March 31, 1949.

THERE is a general impression among clinicians and pathologists that the malignancy of cancer is greater in relatively younger people, and there is certainly a strong belief that cancer occurring in the very old may be so slow in growth as not to limit the "natural" duration of life.

In an attempt to find out to what extent these impressions are justified we shall give:

(*a*) The opinions put forward in some relevant articles in the literature.

(*b*) The results of a study of the microscopic structure of some tumours in patients of different age groups.

Most diseases have an age period of maximum frequency and probably also an age period of maximum intensity and virulence. It does not follow, of course, that these age periods coincide, although it would be reasonable to expect that the age group in which the disease occurred most frequently was also that in which the disease occurred most virulently.

Cancer is predominantly a disease of senescence, and if one had, *a priori*, to foretell its malignancy, one would expect its virulence to be most intense at higher age periods.

On the other hand, the cancer cell seems to differ from the physiological cell only in its greatly increased growth potential. It seems otherwise to share, perhaps disproportionately, in the metabolic turnover of the body. One might expect, since the nutritive efficiency is presumptively greater in the young, that the cancer cells would grow and divide rather faster in the young.

In actual fact the age incidence of disease by frequency and by virulence has not much law or reason, and is just an empirical fact.

We have little doubt that in so far as the belief in increased virulence of cancer at young ages is not based on clinical experience it is based on the assumption that physiological tissue growth and activity is much greater in the young, as, for example, in the muscular hyperplasia following functional over-use.

### RELEVANT LITERATURE.

The malignancy of any tumour can be measured clinically in two ways—by the duration of the untreated disease, and by the curability rate after standard treatment.

Opportunities for studying the first are fortunately diminishing, and one is largely dependent on the classical work of Lazarus-Barlow and Leeming (1924) and of Wyard (1925). Greenwood (1926) gave tables of figures from the data of these and other workers showing the mean natural duration by age groups for untreated cancer of breast, cervix uteri, rectum, larynx, oesophagus, stomach, tongue and mouth (males). He commented (p. 15):

> "It will be seen that either there is no significant relation between age at onset and duration or, at the least, these data are not numerous enough to establish it"; and again (p. 15):

> ". . . on the testimony of these data we cannot confirm the general opinion of clinicians that age at onset is an important factor of duration" (i.e. duration of life with untreated cancer).

There are two other fairly large series of cases of untreated cancer.

Daland (1927) described the course of 100 cases of untreated cancer of the breast. At one place (p. 266) he said that "cancer of the breast is usually very malignant in a young woman," but his figures show no definite correlation between duration of survival and age.

Daland, Welch and Nathanson (1936) analysed the findings in 100 cases of untreated rectal cancer and tabulated their results. They commented (p. 453):

> "It will be observed from the table that the age of the patient bears no relation to the duration of the disease."

Some opinions regarding age-malignancy correlations in different series of treated carcinomas at varying sites are given below.

*Breast.*

Sistrunk and MacCarty (1922, p. 62):

> "Age seems to have a definite bearing on the results to be expected following operation. Forty-one and seven-tenths per cent of the patients over fifty are alive from five to eight years after operation, while only 31·8 per cent of those under fifty have lived a corresponding time."

Stout (1932, p. 301):

> "For the large majority of breast cancers the age of the woman has little significance, but for the extremes prognosis is definitely affected in group statistics by the age at time of operation. In our experience below the age of forty years axillary metastasis is much more apt to be present at the time of operation than beyond that age. In the same way postoperative survivals, as a whole, and apparently cancer-free survivals are decidedly fewer below the age of thirty-five years and decidedly greater beyond the age of sixty-nine years."

Nathanson and Welch (1936, p. 52):

> "The median length of life in the age group below forty is about three years, from forty to sixty about three and a half years, and about four years thereafter. Cancer of the breast in the hospital cases is more malignant in the young and less so in the old."

Scarff and Handley (1938, p. 583):

> "It has been current teaching that carcinoma occurring in a young woman kills more rapidly than in an old woman. Our investigation has not borne this out and Table III shows that age has little bearing on prognosis. No significant variation has been found in the proportion of cases showing axillary involvement in the various age-groups."

Macdonald (1942, p. 76):

"The evidence presented here strongly supports the contention that the best results in the treatment of breast cancer are obtained between the ages of 35 and 50. The thesis that an especial virulence exists for mammary carcinoma in the young is not verified."

Haagensen and Stout (1942) studied the data in 1040 cases of carcinoma of the breast. In 640 of these cases radical mastectomy was carried out. Of these 640 patients, 18 were aged less than 30 years. These authors commented (p. 860):

"These data leave no doubt that radical mastectomy is worth while, even in the youngest patients with breast carcinoma, provided, of course, that the lesion itself is not too far advanced. If the three patients under the age of 30 in our series whose tumor developed during pregnancy or lactation are excluded . . . the five-year cure rate in the remaining 15 young patients rises to 37·5 per cent, a figure quite comparable with the cure rate for our series of cases as a whole.

"Indeed, when the patients in our series are grouped according to age into broader categories there is surprisingly little difference in the results of operation in the different groups." (The age groups were, Under 45, 45 to 49, and 50 or over.)

de Cholnoky (1943, p. 60):

"The conclusion is reached that the results of radical surgery in young women under 30 years of age are comparable to those obtained in the more advanced age groups. The previously held belief that the prognosis for women under 30 years of age who have malignant tumors of the breast is fatal seems untenable."

Hawkins (1943, p. 456):

"Although it is difficult to weigh properly all the various dependent variables involved, the age of the patient does not significantly influence the over-all results of breast-cancer therapy."

Geschickter (1945, p. 404):

"Thus, while the number of five-year survivals in the younger age group compares favorably with those in the older group, the average duration of life for both cured and non-cured patients is less in the younger group. This is because of the rapid termination of fatal tumors in the young, particularly during pregnancy and lactation."

*Rectum.*

Fowler (1926, p. 77):

"It cannot be said, therefore, that the presence or absence of involvement of the lymph nodes in rectal carcinoma in the young is of any prognostic value as far as recovery is concerned. However, a higher percentage of involvement of lymph nodes is found, and the average postoperative life is shorter (16·7 months) than in similar cases in the adult."

Dukes (1940) found that patients under the age of 40 years had a higher incidence of lymphatic metastases than those in the age group 40–59 years. He commented (p. 529):

"The most likely explanation is that cancer of the rectum gives rise to metastases more rapidly in young patients than in old."

*Uterus (cervix).*

Lane-Claypon (1927, p. 34):

"The evidence therefore does not show any tendency to greater virulence or apparently to a more rapid dissemination of the growth in youth."

*Lung.*

Geschickter and Denison (1934) analysed 60 cases of primary lung cancer and graded them histologically, correlating the histological findings with clinical malignancy and with age. They gave their opinion that the most malignant forms, both clinically and histologically, tended to occur at the younger ages.

Frissell and Knox (1937) gave details of 46 cases of primary lung cancer which included the age and the duration of the disease for each patient. On examining these data we have found that there is no relation at all between age and the duration of the disease. The authors stated (p. 225):

"These figures are sufficient to indicate the rarity of carcinoma of the lung under the age of forty. It is, however, characterized by the same clinical and pathological course in this age group as in more elderly persons."

The following opinions have been put forward more particularly with reference to malignancy as assessed histologically.

### *Carcinoma in General.*

Fowler (1926) analysed the findings in 112 cases of malignant epithelial neoplasms occurring in persons under 26 years of age. He divided them into a "carcinoma" group of 89 patients and an "epithelioma" group of 23 patients. He had no doubt that the clinical malignancy of the tumours in the carcinoma group was greater in these patients than in older patients, and he analysed the histological findings to see whether the histological malignancy ran parallel. He stated (p. 74):

"Compared with sections of similar tumors in older persons, the tumors in these cases give a microscopic picture of a more rapidly growing, undifferentiated tissue, often with many mitotic figures in each field."

He found that hyalinization was never present; that lymphocytic infiltration and fibrosis were present to a moderate degree and only in the less malignant tumours; and that cellular differentiation was present mainly in ovarian and thyroid carcinomas where, in each instance, the mortality was comparatively low. He concluded (p. 83):

"The lack of hyalinization, fibrosis, lymphocytic infiltration, and cellular differentiation may have been responsible for the increased malignancy of these neoplasms in the young."

*Rectum.*

Dukes (1940) divided his cases of rectal carcinoma into histological grades. He found (p. 532):

"The distribution of the grades was approximately the same in each sex, but tumours of high-grade malignancy appeared to be more common in young patients, though the difference did not quite reach the accepted level of significance in the $\chi^2$ test."

*Lung.*

As mentioned earlier, Geschickter and Denison (1934) analysed 60 cases of primary lung carcinoma. One of their conclusions was (p. 875):

"Hilar or epidermoid carcinoma may be graded into three forms. In the least malignant histological grade, occurring in patients over fifty years, squamous cells predominate. The middle grade shows a proliferation of basal and transitional cells and occurs in patients under fifty years. The most malignant grade shows masses of compact or spindle cells, is referred to as oat-cell cancer, and is most common in young adults. Lobular adenocarcinoma is also divided histologically into three groups. The least malignant or most highly differentiated tumors are of two forms, adeno-columnar and adenomucoid. The average age of these patients is forty years. The most malignant histologic type of adenocarcinoma shows a diffuse proliferation of cuboidal cells (adenocuboidal cancer). The patients affected are most often under forty years of age and sometimes under thirty."

Frissell and Knox (1937) gave the histological diagnosis of the type of growth in all but three of their 46 cases of primary lung carcinoma. Analysis of their data shows no significant association of the least differentiated tumours with the younger patients.

*Stomach.*

With regard to the 9 cases of gastric carcinoma included in his series, Fowler (1926) commented (p. 78):

"The histological picture is that of a rapidly growing adenocarcinoma, the cells in some parts retaining their glandular arrangement, in others forming long cords or solid groups of undifferentiated cells. . . .

"This pathological appearance is borne out by the fact that death occurred within six months of the date of operation in all cases."

It will be seen from these quotations that opinions vary on the subject of the relation of age to both clinical and histological malignancy. Certainly for the breast opinion seems to have veered during the last decade to a denial of increased malignancy at younger ages.

Ewing (1940) commented on the matter as follows (p. 17):

"The weight of opinion indicates that adult types of tumors occurring at early ages tend to run a more rapid course, to metastasize more widely, but this view is contested by many observers."

Since there is such diversity of opinion as to whether this effect exists at all it is reasonable to assume that if it does exist it is small.

### ASSESSMENT OF MICROSCOPIC APPEARANCES OF CARCINOMAS AT DIFFERENT AGES.

Our material has been taken from the collection of microscopic sections of tumours in this laboratory.

For many years it has been the rule that when a neoplasm was diagnosed during routine reporting it was recorded in classifications by site and by type. It frequently happened, of course, that the diagnosis was in doubt. When this happened the tumour was recorded once, and once only, under its most likely diagnosis although a note of it might be made for reference under several headings.

Since there is no reason to suspect that there was any systematic bias towards diagnosing malignancy unduly at one age group rather than another, comparison of random samples of the tumour sections from different age groups is statistically valid.

*Procedure.*

We went through the index cards of the years 1930 to 1948. Whenever a case of carcinoma was found occurring at age 30 or under in any of uterus, lip, lung, stomach, colon, rectum or breast, we carried on through the cards and chose the first carcinoma recorded in the same organ and of the same sex in an individual in age group 46–55 and again in a high age group. Due to the varying rarity of cancer at high ages the high age groups chosen were :

75 years and over for breast ;

70 years and over for uterus, lip, stomach, colon, rectum ; and

61 years and over for lung (where the middle age group was 41–50).

For each tumour type we thus had three randomly chosen groups of slides, of equal numbers, occurring in the young, the middle-aged and the old. The slides were then taken out of their holders and shuffled. This made identification of the sections impossible without reference to the laboratory register of the numbers etched on the slides. The shuffled and randomized sections were then examined for some or all of certain characteristics :

Degree of epidermoid or glanduliform differentiation.

Degree of fibrosis.

Cell size and polymorphism.

Amount of " colloid " formation.

Mitotic frequency.

General impression of malignancy.

All these features were given one of four grades of increasing expression.

For *cell size* (which in this context means cell largeness), *cell polymorphism* and *degree of fibrosis* (scirrhous reaction) the principle adopted was to allocate Grades I and IV to cases which were strikingly low and strikingly high respectively in these features. Grades II and III formed intermediate groups of " low moderate " and " high moderate " respectively.

For *epidermoid differentiation* the scale, as well as could be defined, was :

Grade I. No differentiation.

,, II. Formation of solid cell masses with a demarcated cellular border, i.e. a " solid alveolar " pattern.

,, III. Formation of prickle cells and squamous cells.

,, IV. Formation of definite keratinized " pearls."

*Glanduliform differentiation* varied from no differentiation in Grade I, upwards, with increasingly glanduliform pattern, through Grades II, III and IV,

Carcinomas of colon and rectum usually show relatively much glandulifrom differentiation. At these sites Grade I meant no or notably little glanduliform differentiation.

*"Colloid" formation.*—In this feature Grade I applies to cases showing no "colloid" formation. Grades II, III and IV indicate increasing proportions of this degeneration.

*Mitotic frequency.*—Tumour types tend to have a characteristic mitotic frequency. The gradings used for the various organs were :

| | Uterus, rectum, colon. | Breast, stomach. | Lip. |
|---|---|---|---|
| Grade I | 0–1 | 0–1 | 0–2 |
| ,, II | 2–3 | 2 | 3 |
| ,, III | 4–5 | 3 | 4 |
| ,, IV | 6 and + | 4 and + | 5 and + |

In each case the figures refer to the average number of mitoses per 1/6 in. field. In scirrhous tumours allowance was made for the space occupied by fibrous tissue.

When all the sections had been examined and the various factors assessed the slides were identified by reference to the laboratory register and the results then tabulated according to the three age groups. The results are shown in Table I.

TABLE I.—*Distinguishing Age, Site of Carcinoma and Grade of Degree, Intensity or Extent of Each Feature Estimated. (The figures give the number of cases occurring within each group.)*

| | Number of cases. | | Epidermoid differentiation. | | | | Glanduliform differentiation. | | | | Scirrhous reaction. | | | |
|---|---|---|---|---|---|---|---|---|---|---|---|---|---|---|
| *Age 30 years or less.* | M. | F. | I. | II. | III. | IV. | I. | II. | III. | IV. | I. | II. | III. | IV. |
| Carcinoma of— | | | | | | | | | | | | | | |
| (*a*) Uterus | .. | 20* | 3 | 7 | 6 | 0 | 1 | 2 | 1 | 0 | 5 | 3 | 11 | 21 |
| (*b*) Lip | 9 | 1 | 0 | 0 | 5 | 5 | .. | .. | .. | .. | .. | .. | .. | .. |
| (*c*) Lung | 20 | 2 | 14 | 6 | 2 | 0 | 21 | 0 | 1 | 0 | .. | .. | .. | .. |
| (*d*) Stomach | 2 | 6 | .. | .. | .. | .. | 4 | 4 | 0 | 0 | 0 | 1 | 6 | 1 |
| (*e*) Colon | 3 | 3 | .. | .. | .. | .. | 2 | 1 | 2 | 1 | 1 | 2 | 3 | 0 |
| (*f*) Rectum | 7 | 7 | .. | .. | .. | .. | 3 | 5 | 5 | 1 | 1 | 4 | 4 | 5 |
| (*g*) Breast | .. | 25 | .. | .. | .. | .. | 16 | 1 | 7 | 1 | 11 | 11 | 3 | 0 |
| Total | 41 | 64 | 17 | 13 | 13 | 5 | 47 | 13 | 16 | 3 | 18 | 21 | 27 | 27 |
| *Age 46–55 years.* | | | | | | | | | | | | | | |
| Carcinoma of : | | | | | | | | | | | | | | |
| (*a*) Uterus | .. | 20* | 1 | 8 | 5 | 2 | 0 | 1 | 3 | 0 | 4 | 4 | 9 | 3 |
| (*b*) Lip | 9 | 1 | 0 | 2 | 4 | 4 | .. | .. | .. | .. | .. | .. | .. | .. |
| (*c*) Lung (41–50 yrs.) | 20 | 2 | 7 | 6 | 8 | 1 | 20 | 2 | 0 | 0 | .. | .. | .. | .. |
| (*d*) Stomach | 2 | 6 | .. | .. | .. | .. | 3 | 2 | 2 | 1 | 1 | 2 | 4 | 1 |
| (*e*) Colon | 3 | 3 | .. | .. | .. | .. | 0 | 2 | 4 | 0 | 0 | 1 | 5 | 0 |
| (*f*) Rectum | 7 | 7 | .. | .. | .. | .. | 0 | 3 | 8 | 3 | 1 | 4 | 9 | 0 |
| (*g*) Breast | .. | 25 | .. | .. | .. | .. | 16 | 4 | 5 | 0 | 8 | 8 | 9 | 0 |
| Total | 41 | 64 | 8 | 16 | 17 | 7 | 39 | 14 | 22 | 4 | 14 | 19 | 36 | 4 |
| *Age 70 years or more.* | | | | | | | | | | | | | | |
| Carcinoma of : | | | | | | | | | | | | | | |
| (*a*) Uterus | .. | 20* | 6 | 7 | 1 | 2 | 0 | 1 | 1 | 2 | 6 | 1 | 12 | 1 |
| (*b*) Lip | 9 | 1 | 1 | 3 | 5 | 1 | .. | .. | .. | .. | .. | .. | .. | .. |
| (*c*) Lung (61 y. and over) | 20 | 2 | 8 | 3 | 7 | 4 | 22 | 0 | 0 | 0 | .. | .. | .. | .. |
| (*d*) Stomach | 2 | 6 | .. | .. | .. | .. | 1 | 1 | 3 | 3 | 2 | 5 | 1 | 0 |
| (*e*) Colon | 3 | 3 | .. | .. | .. | .. | 2 | 0 | 3 | 1 | 0 | 0 | 5 | 1 |
| (*f*) Rectum | 7 | 7 | .. | .. | .. | .. | 0 | 4 | 5 | 5 | 1 | 3 | 9 | 1 |
| (*g*) Breast (75 y. and over) | .. | 25 | .. | .. | .. | .. | 15 | 3 | 4 | 3 | 13 | 6 | 6 | 0 |
| Total | 41 | 64 | 15 | 13 | 13 | 7 | 40 | 9 | 16 | 14 | 22 | 15 | 33 | 3 |

* 16 cervical and 4 corporeal carcinomas.

TABLE I—(*cont.*).

| | Number of cases. M. | Number of cases. F. | Cell size. I. | Cell size. II. | Cell size. III. | Cell size. IV. | Cell polymorphism. I. | Cell polymorphism. II. | Cell polymorphism. III. | Cell polymorphism. IV. | Mitotic frequency. I. | Mitotic frequency. II. | Mitotic frequency. III. | Mitotic frequency. IV. |
|---|---|---|---|---|---|---|---|---|---|---|---|---|---|---|
| ***Age* 30 *years or less.*** | | | | | | | | | | | | | | |
| Carcinoma of: | | | | | | | | | | | | | | |
| (*a*) Uterus | .. | 20* | .. | .. | .. | .. | .. | .. | .. | .. | 4 | 12 | 4 | 0 |
| (*b*) Lip | 9 | 1 | .. | .. | .. | .. | .. | .. | .. | .. | 8 | 1 | 0 | 1 |
| (*c*) Lung | 20 | 2 | 6 | 11 | 4 | 1 | 2 | 11 | 8 | 1 | .. | .. | .. | .. |
| (*d*) Stomach | 2 | 6 | .. | .. | .. | .. | .. | .. | .. | .. | 3 | 3 | 2 | 0 |
| (*e*) Colon | 3 | 3 | .. | .. | .. | .. | .. | .. | .. | .. | 2 | 3 | 1 | 0 |
| (*f*) Rectum | 7 | 7 | .. | .. | .. | .. | .. | .. | .. | .. | 5 | 7 | 2 | 0 |
| (*g*) Breast | .. | 25 | 1 | 8 | 13 | 3 | .. | .. | .. | .. | 8 | 9 | 6 | 2 |
| Total | 41 | 64 | 7 | 19 | 17 | 4 | 2 | 11 | 8 | 1 | 30 | 35 | 15 | 3 |
| ***Age* 46–55 *years.*** | | | | | | | | | | | | | | |
| Carcinoma of: | | | | | | | | | | | | | | |
| (*a*) Uterus | .. | 20* | .. | .. | .. | .. | .. | .. | .. | .. | 9 | 7 | 4 | 0 |
| (*b*) Lip | 9 | 1 | .. | .. | .. | .. | .. | .. | .. | .. | 8 | 0 | 2 | 0 |
| (*c*) Lung (41–50 y.) | 20 | 2 | 3 | 9 | 10 | 0 | 1 | 14 | 5 | 2 | .. | .. | .. | .. |
| (*d*) Stomach | 2 | 6 | .. | .. | .. | .. | .. | .. | .. | .. | 1 | 5 | 2 | 0 |
| (*e*) Colon | 3 | 3 | .. | .. | .. | .. | .. | .. | .. | .. | 1 | 2 | 3 | 0 |
| (*f*) Rectum | 7 | 7 | .. | .. | .. | .. | .. | .. | .. | .. | 3 | 6 | 5 | 0 |
| (*g*) Breast | .. | 25 | 0 | 9 | 15 | 1 | .. | .. | .. | .. | 9 | 11 | 1 | 4 |
| Total | 41 | 64 | 3 | 18 | 25 | 1 | 1 | 14 | 5 | 2 | 31 | 31 | 17 | 4 |
| ***Age* 70 *years or more.*** | | | | | | | | | | | | | | |
| Carcinoma of: | | | | | | | | | | | | | | |
| (*a*) Uterus | .. | 20* | .. | .. | .. | .. | .. | .. | .. | .. | 14 | 5 | 1 | 0 |
| (*b*) Lip | 9 | 1 | .. | .. | .. | .. | .. | .. | .. | .. | 5 | 4 | 1 | 0 |
| (*c*) Lung (61 y. and over) | 20 | 2 | 2 | 8 | 12 | 0 | 4 | 9 | 8 | 1 | .. | .. | .. | .. |
| (*d*) Stomach | 2 | 6 | .. | .. | .. | .. | .. | .. | .. | .. | 1 | 3 | 1 | 3 |
| (*e*) Colon | 3 | 3 | .. | .. | .. | .. | .. | .. | .. | .. | 4 | 2 | 0 | 0 |
| (*f*) Rectum | 7 | 7 | .. | .. | .. | .. | .. | .. | .. | .. | 3 | 4 | 6 | 1 |
| (*g*) Breast (75 y. and over) | .. | 25 | 0 | 13 | 9 | 3 | .. | .. | .. | .. | 12 | 8 | 4 | 1 |
| Total | 41 | 64 | 2 | 21 | 21 | 3 | 4 | 9 | 8 | 1 | 39 | 26 | 13 | 5 |

| | Number of cases. M. | Number of cases. F. | "Colloid" formation. I. | "Colloid" formation. II. | "Colloid" formation. III. | "Colloid" formation. IV. | General malignancy. I. | General malignancy. II. | General malignancy. III. | General malignancy. IV. |
|---|---|---|---|---|---|---|---|---|---|---|
| ***Age* 30 *years or less.*** | | | | | | | | | | |
| Carcinoma of: | | | | | | | | | | |
| (*a*) Uterus | .. | 20* | .. | .. | .. | .. | 1 | 4 | 13 | 2 |
| (*b*) Lip | 9 | 1 | .. | .. | .. | .. | 3 | 6 | 0 | 1 |
| (*c*) Lung | 20 | 2 | .. | .. | .. | .. | 1 | 6 | 11 | 4 |
| (*d*) Stomach | 2 | 6 | 7 | 0 | 1 | 0 | 0 | 4 | 4 | 0 |
| (*e*) Colon | 3 | 3 | 2 | 0 | 2 | 2 | 0 | 3 | 3 | 0 |
| (*f*) Rectum | 7 | 7 | 8 | 2 | 4 | 0 | 1 | 5 | 5 | 3 |
| (*g*) Breast | .. | 25 | 25 | 0 | 0 | 0 | 4 | 7 | 11 | 3 |
| Total | 41 | 64 | 42 | 2 | 7 | 2 | 10 | 35 | 47 | 13 |
| ***Age* 46–55 *years.*** | | | | | | | | | | |
| Carcinoma of: | | | | | | | | | | |
| (*a*) Uterus | .. | 20* | .. | .. | .. | .. | 6 | 8 | 5 | 1 |
| (*b*) Lip | 9 | 1 | .. | .. | .. | .. | 4 | 5 | 1 | 0 |
| (*c*) Lung (41–50 y.) | 20 | 2 | .. | .. | .. | .. | 2 | 14 | 5 | 1 |
| (*d*) Stomach | 2 | 6 | 8 | 0 | 0 | 0 | 0 | 3 | 5 | 0 |
| (*e*) Colon | 3 | 3 | 6 | 0 | 0 | 0 | 0 | 1 | 5 | 0 |
| (*f*) Rectum | 7 | 7 | 14 | 0 | 0 | 0 | 1 | 6 | 6 | 1 |
| (*g*) Breast | .. | 25 | 25 | 0 | 0 | 0 | 3 | 10 | 10 | 2 |
| Total | 41 | 64 | 53 | 0 | 0 | 0 | 16 | 47 | 37 | 5 |
| ***Age* 70 *years or more.*** | | | | | | | | | | |
| Carcinoma of: | | | | | | | | | | |
| (*a*) Uterus | .. | 20* | .. | .. | .. | .. | 5 | 7 | 6 | 2 |
| (*b*) Lip | 9 | 1 | .. | .. | .. | .. | 1 | 6 | 2 | 1 |
| (*c*) Lung (61 y. and over) | 20 | 2 | .. | .. | .. | .. | 3 | 10 | 7 | 2 |
| (*d*) Stomach | 2 | 6 | 8 | 0 | 0 | 0 | 1 | 1 | 6 | 0 |
| (*e*) Colon | 3 | 3 | 5 | 0 | 1 | 0 | 1 | 2 | 2 | 1 |
| (*f*) Rectum | 7 | 7 | 11 | 0 | 3 | 0 | 1 | 5 | 8 | 0 |
| (*g*) Breast (75 y. and over) | .. | 25 | 23 | 0 | 2 | 0 | 7 | 9 | 8 | 1 |
| Total | 41 | 64 | 47 | 0 | 6 | 0 | 19 | 40 | 39 | 7 |

* 16 cervical and 4 corporeal carcinomas.

*Analysis of the Tables.*

We started this experiment with the hypotheses :

(*a*) That there was no correlation between age and any of the factors examined.

(*b*) That if there was a correlation between age and any factor we had no reason to expect the correlation with one organ or one factor more than another.

The analysis of the figures therefore resolves itself into an estimation of the probability with which these figures would occur by chance if these hypotheses were true.

The collection of the data was designed so that we should be able to extract the effects due to (*a*) age (in three blocks—youth, middle age, old age), and (*b*) any change in the threshold for diagnosis of malignancy or in the type of material sent to the laboratory between 1930 and 1948, the period covered by the material.

This was done by balancing each unit in age group 30 and under by a randomly selected unit in each of the other age groups diagnosed at about the same time. After completing the experiment, we were quite convinced that there was no such gross systematic change in type of material or standards of diagnosis over the years. We have, therefore, not considered that the difference between blocks arranged by date of diagnosis would appreciably reduce the residual error on which the significance of inter-age differences would be estimated.

The experiment was therefore designed to allow for an analysis of variance. For example, in the sub-experiment " degree of glanduliform differentiation in rectal carcinoma " there was a total of 42 units or slides divided into 3 groups of 14 each for patients of 30 years or less, 46 to 55 years, and 70 years or more respectively, and divisible also into 14 groups of 3 each, 1 in each age group, diagnosed in the same calendar year. In an analysis of the variance in this balanced design there would then have been a total of 41 " degrees of freedom " comprising 2 for age, 13 for date of diagnosis, and 26 for residual error. Of the 2 degrees of freedom for age, one could have been used to test the linearity of the difference by age if necessary. In fact there was no need for this since a relatively coarse analysis of the data indicated that the figures might well have occurred by chance —on the hypothesis that there was no correlation between age and any of the factors we estimated.

The $\chi^2$ test was carried out for each factor, with the exception of " ' colloid ' formation " and " cell polymorphism," using the total figures of each factor for each age group. For example, in the case of " epidermoid differentiation " there was a contingency table of the form—

| | | | |
|---|---|---|---|
| 17 | 13 | 13 | 5 |
| 8 | 16 | 17 | 7 |
| 15 | 13 | 13 | 7. |

The results for the various factors were :

*Epidermoid differentiation* :

$$n = 6 \quad \chi^2 = 4{\cdot}95 \quad P = 0{\cdot}7\text{–}0{\cdot}5.$$

*Glanduliform differentiation* (consolidating Grades III and IV) :

$$n = 4 \quad \chi^2 = 4{\cdot}55 \quad P = c.\ 0{\cdot}3.$$

*Scirrhous reaction* (consolidating Grades III and IV) :

$$n = 4 \quad \chi^2 = 3{\cdot}30 \quad P. = c.\ 0{\cdot}5.$$

*Cell size* (consolidating Grades I and II, and Grades III and IV) :

$$n = 2 \quad \chi^2 = 1{\cdot}08 \quad P = 0{\cdot}7\text{–}0{\cdot}5.$$

*Mitotic frequency* (consolidating Grades III and IV) :

$$n = 4 \quad \chi^2 = 3{\cdot}12 \quad P = c.\ 0{\cdot}5.$$

*General malignancy* :

$$n = 6 \quad \chi^2 = 10{\cdot}06 \quad P = 0{\cdot}2\text{–}0{\cdot}1.$$

It will be seen that in no case are the figures " significantly " different from what might be expected by chance. Inspection of Table I does indeed suggest that the figures for epidermoid differentiation in bronchogenic carcinoma might well give a significant correlation between increasing age and increasing degree of epidermoid differentiation. We have carried out a $\chi^2$ test for epidermoid differentiation in bronchogenic carcinoma alone consolidating the figures for Grades II, III and IV. This gives $n = 2$ $\chi^2 = 5{\cdot}29$ $P = 0{\cdot}05$ ($P_{\cdot 05} = 5{\cdot}991$ $P_{\cdot 02} = 7{\cdot}824$).

Since this is only one among 30 (not completely independent) sub-experiments we could, of course, expect the figures for one or more of these sub-experiments to show what would have been by itself a probability of 1 in 20. At the beginning of the experiment, however, we laid down the hypothesis that we had no reason to expect significant results from one tumour type or characteristic more than another. It follows that there is, so far as our results *per se* are concerned, no reason to regard this as indicating a real correlation between age and increasing epidermoid differentiation in bronchogenic carcinoma. It is notable, however, that in our reading of the literature the tumour in which the correlation between increasing differentiation and increasing age has been most strikingly demonstrated (Geschickter and Denison, 1934) is also bronchogenic carcinoma. Consequently our figures, in so far as they are confirmatory, are also suggestive.

We did, in fact, although we have not given the table, estimate, using the method of analysis of variance, the probability with which the figures for epidermoid differentiation and age could have occurred by chance for epidermoid differentiation of (*a*) bronchogenic carcinoma, (*b*) carcinoma of the cervix, (*c*) carcinoma of lip. The probabilities were, respectively, c. 0·01 ; not significant ; c. 0·05. In carcinoma of the lip and in carcinoma of the lung the sense of the association, although in each case " significant," was unfortunately opposite, i.e. as judged by each set of figures taken separately, carcinomas of the lung tend to be more differentiated at higher ages, and carcinomas of the lip less differentiated at higher ages.

We can summarize our findings thus far in the simple statement that there is no good evidence of a correlation between the age of the patient and the degree of any of the histological factors we have estimated, with the possible exception that carcinomas of the lung tend to be undifferentiated in younger patients.

In order to investigate this matter further we made another selection of slides from the files, all examples of bronchogenic carcinoma, all chosen and examined in the same way, using the same criteria. These slides did not include any of those already used. They comprised 216 sections, 54 sections in each of the age groups 31–40, 41–50, 51–60, and 61 and over. Of the 54 patients in each group, 46 were male and 8 female.

The results are given in Table II. They were analysed by estimating the significance of the slope of the regression line of increasing epidermoid differentiation on increasing age.

TABLE II.—*Distinguishing Degree of Epidermoid Differentiation as Found in* 216 *Carcinomas of the Lung,* 54 *in Each of Four Age Groups*—31–40 *years,* 41–50 *years,* 51–60 *years and* 61 *years and over—giving the Number of Cases in Each Grade in Each Age Group.*

| | 31–40. | 41–50. | 51–60. | 61 and over. |
|---|---|---|---|---|
| Grade I | 40 | 36 | 33 | 33 |
| Grade II | 6 | 13 | 8 | 12 |
| Grade III | 4 | 4 | 10 | 8 |
| Grade IV | 4 | 1 | 3 | 1 |

The regression line was found by using the formula—

$$y - \bar{y} = b(x - \bar{x})$$

where $y$ is the grade of epidermoid differentiation found, $x$ is the age in years, and $b = \frac{\Sigma(y - \bar{y})(x - \bar{x})}{\Sigma(x - \bar{x})^2}$.

$b$, the slope of the best fitting regression line, was found to be 0·0052. The variance of $b$, using the formula—

$$V_b = \frac{1}{(n-2)\,\Sigma(x - \bar{x})^2}\left[\Sigma(y^2) - \frac{(\Sigma y)^2}{n} - \frac{[\Sigma y(x - \bar{x})]^2}{\Sigma(x - \bar{x})^2}\right]$$

was found to be 0·000028139. This means that figures which give a regression line with this small slope might easily have occurred by chance, and there is no significant correlation between increasing age and increasing degree of epidermoid differentiation shown by these figures.

It is to be noted, however, that the range of age was small since there were no more sections from patients aged 30 years or less available for study, and it might well be that it is the patients under 30 who give the curve its significance.

In summary we would say that there is a case for the existence of such a correlation though it cannot be very strong.

### DISCUSSION.

Opinions in the literature on the subject are contradictory. Certainly there is no consensus of opinion that the malignancy of cancer as estimated clinically is greater in younger patients than in older patients. As far as we can estimate there is certainly no difference histologically. How far histological appearances are themselves correlated with growth rate and malignant behaviour in a carcinoma is, of course, a debatable matter, but in so far as they are an indication of the degree of clinical malignancy the evidence derived from them does not confirm the belief that tumours are more malignant at young ages.

We think that the data we have given add up to the statement that carcinoma at younger ages is not more malignant than at older ages, or at least that the contrary has not been proved.

### SUMMARY.

The histological appearances of carcinomas from 105 patients aged 30 years or less have been compared with those of a similar number of patients in age groups 46–55 (41–50 for lung) and 70 and over (61 and over for lung). No significant differences have been found except doubtfully in carcinoma of the lung.

W. W. Park is in receipt of a grant from the British Empire Cancer Campaign.

### REFERENCES.

DALAND, E. M.—(1927) *Surg. Gynec. Obstet.*, **44**, 264.

*Idem*, WELCH, C. E., AND NATHANSON, I.—(1936) *New Engl. J. Med.*, **214**, 451.

DE CHOLNOKY, T.—(1943) *Surg. Gynec. Obstet.*, **77**, 55.

DUKES, C. E.—(1940) *J. Path. Bact.*, **50**, 527.

EWING, J.—(1940) in 'Cancer in Childhood,' edited by Dargeon, H. W. London (Kimpton).

FOWLER, L. H. (1926)—*Surg. Gynec. Obstet.*, **43**, 73.

FRISSELL, L. F., AND KNOX, L. C.—(1937) *Amer. J. Cancer*, **30**, 219.

GESCHICKTER, C. F.—(1945) 'Diseases of the Breast,' 2nd Ed. Philadelphia (J. B. Lippincott Co.).

*Idem* AND DENISON, R.—(1934) *Amer. J. Cancer*, **22**, 854.

GREENWOOD, M.—(1926) *Reports on Public Health and Medical Subjects*, No. 33. London (H.M. Stationery Office).

HAAGENSEN, C. D., AND STOUT, A. P.—(1942) *Ann. Surg.*, **116**, 801, and **118**, 859, 1032.

HAWKINS, J. W.—(1943) *J. nat. Cancer Inst.*, **4**, 445.

LANE-CLAYPON, J. E.—(1927) *Reports on Public Health and Medical Subjects*, No. 40. London (H.M. Stationery Office).

LAZARUS-BARLOW, W. S., AND LEEMING, J. H.—(1924) *Brit. med. J.*, **2**, 266.

MACDONALD, I.—(1942) *Surg. Gynec, Obstet.*, **74**. 75.

NATHANSON, I. T., AND WELCH, C. E.—(1936) *Amer. J. Cancer*, **28**, 40.

SCARFF, R. W., AND HANDLEY, R. S.—(1938) *Lancet*, ii, 582.

SISTRUNK, W. E., AND MACCARTY, W. C.—(1922) *Ann. Surg.*, **75**, 61.

STOUT, A. P.—(1932) 'Human Cancer.' London (Kimpton).

WYARD, S.—(1925) *Brit. med. J.*, i, 206.